# Supplementary material for: Discovery and pharmacophoric characterization of chemokine network inhibitors using phage-display, saturation mutagenesis and computational modelling
Source: Nat Commun. 2023 Sep 16;14:5763. doi: 10.1038/s41467-023-41488-z (PMC10505172; doi:10.1038/s41467-023-41488-z)
Supplement: Supplementary file 4 — Source Data [file 41467_2023_41488_MOESM4_ESM.zip › SourceData/scripts/xNNKs_20230808183658.tar!/xNNKs/analysis/heatmaps/SV_HD2X_leucine_heatmap.pdf]

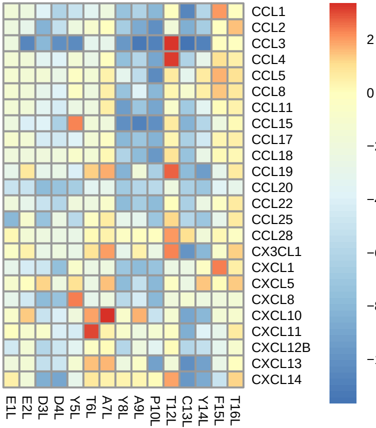
CCL1
CCL2
CCL3
CCL4
CCL5
CCL8
CCL11
CCL15
CCL17
CCL18
CCL19
CCL20
CCL22
CCL25
CCL28
CX3CL1
CXCL1
CXCL5
CXCL8
CXCL10
CXCL11
CXCL12B
CXCL13
CXCL14
E1L
E2L
D3L
D4L
Y5L
T6L
A7L
Y8L
A9L
P10L
T12L
C13L
Y14L
F15L
T16L
2
0
−2
−4
−6
−8
−10
